# Highly Efficient and Genotype‐Independent Genetic Transformation System in Sugarcane

**DOI:** 10.1111/pbi.70486

**Published:** 2025-12-05

**Authors:** Wenzhi Wang, Dongjiao Wang, Wanying Zhao, Yuanyuan Zhang, Zhen Zeng, Yan Hu, Tingting Sun, Linbo Shen, Qibin Wu, Yuebin Zhang, Shuangxia Jin, Shuzhen Zhang, Youxiong Que

**Affiliations:** ^1^ State Key Laboratory of Tropical Crop Breeding Institute of Tropical Bioscience and Biotechnology/Sanya Research Institute, Chinese Academy of Tropical Agricultural Sciences Sanya Hainan China; ^2^ State Key Laboratory of Tropical Crop Breeding Sugarcane Research Institute, Yunnan Academy of Agricultural Sciences Kaiyuan China; ^3^ National Key Laboratory of Crop Genetic Improvement, Huazhong Agricultural University Wuhan China

**Keywords:** callus induction, insect and herbicide resistance, sugarcane, transformation system

Sugarcane (*Saccharum* spp.), a globally important sugar crop, is characterised by a large genome, high heterozygosity and allopolyploid nature, leading to prolonged and inefficient hybrid breeding (Wang, Pan, et al. 2025). Genetic transformation has emerged as a crucial approach for sugarcane genetic improvement and molecular research (Wang, Gou, et al. 2025). However, the existing sugarcane transformation systems suffer from severe genotype dependence, with a narrow range of available recipient materials (Brant et al. 2025). Recently, *Agrobacterium*‐mediated transformation has become the preferred method for sugarcane because of its simplicity, cost‐effectiveness, stable performance and relatively high efficiency (Azizi‐Dargahlou and Pouresmaeil 2024; Brant et al. 2025). Callus is recognised as the optimal material for *Agrobacterium*‐mediated transformation (Gad et al. 2025). To address genotype dependence in sugarcane transformation, eight elite cultivars (ROC22, LC05‐136, GT42, LC1541, YZ08‐1609, YZ05‐51, ZT3 and HPGZ) were selected, and an efficient embryogenic callus system was established. Six vectors harbouring insect‐ and herbicide‐resistant genes were individually introduced into the calli at peak regeneration via *Agrobacterium*. This system supports advancements in sugarcane functional genomics, molecular biology and the development of transgenic plants.

We initially utilised the apical meristem of leaf tissues from eight elite sugarcane cultivars as explants for callus induction. They were transversely sectioned into thin slices and cultured on callus induction media supplemented with varying concentrations of auxin (2,4‐D). Fresh medium was replaced every 15 days over a total cultivation period of 75 days, yielding five successive generations of callus at different growth stages (Figure 1a). Based on the analysis of surface‐cell regeneration values (Figure S1a), the cultivar‐specific optimal 2,4‐D concentrations all fell within 1.0–3.0 mg/L. Across genotypes, 2 mg/L produced the highest overall callus induction and regeneration rates (Figure S1b). Interestingly, when callus induced at genotype‐specific 2,4‐D concentrations were subcultured to the third or fourth subculture prior to differentiation, the optimal duration varied among cultivars. Regeneration efficiency analysis showed that GT42, LC1541, YZ08‐1609, YZ05‐51 and HPGZ reached peak growth status at the third generation (45 days), whereas ROC22, LC05‐136 and ZT3 required a fourth generation (60 days) for optimal performance (Figure S1c). Among these cultivars, ROC22 achieved the highest propagation efficiency, with a single explant producing approximately 8150 plants, whereas HPGZ had the lowest efficiency at approximately 3554 plants per explant (Figure 1b, Figure S2). Detailed protocols for genotype‐specific optimization are provided in Figure S2 and Table S1.

**FIGURE 1:**
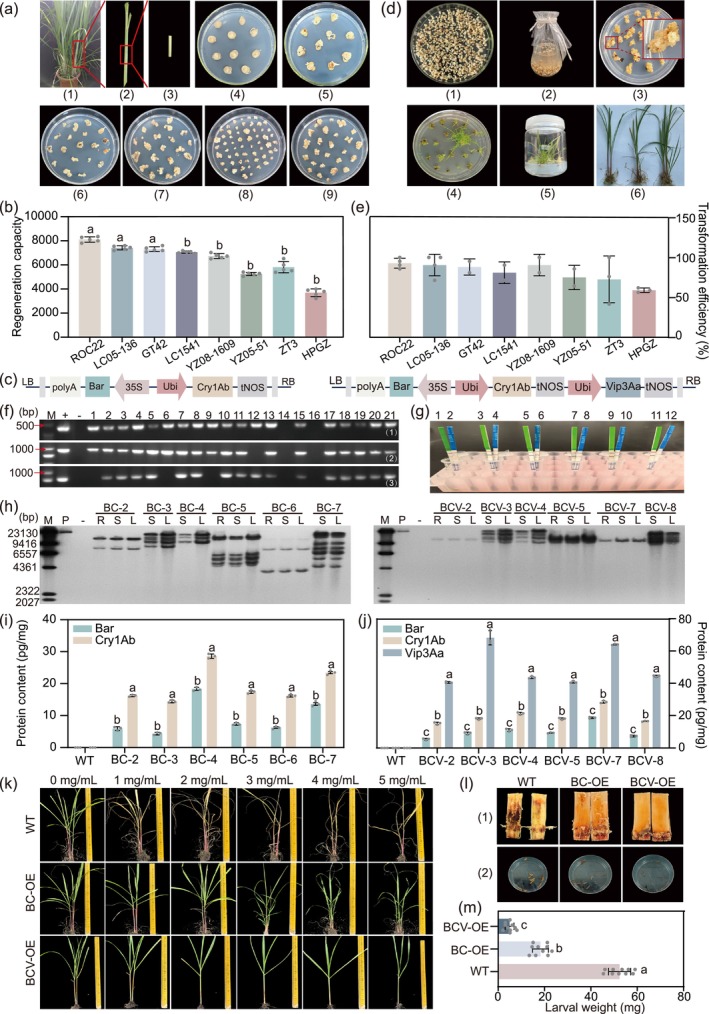
A genotype‐independent transformation system in sugarcane. (a) Somatic embryogenic callus induction. (1–4) Healthy sugarcane plants, untreated explants, immature central whorl leaf tissues and transverse thin sections of explants inoculated on callus induction medium; (5–9) primary, secondary, tertiary, quaternary and quinary callus (days 15, 30, 45, 60 and 75 of induction). (b) Regeneration efficiency. (c) Schematic of plant expression vectors. pBC (left) harbours *Bar* and *Cry1Ab*; pBCV (right) contains *Bar*, *Cry1Ab* and *Vip3Aa*. (d) Genetic transformation. (1) Embryogenic callus collection. (2) *Agrobacterium*‐mediated infection. (3) Resistant callus selection. (4) Differentiated selection. (5) Rooting selection. (6) Acclimatisation and transplanting. (e) Genetic transformation efficiency. (f) PCR detection of transgenic plants carrying the pBCV insect‐resistance vector. (1–3) *Bar*, *Cry1Ab* and *Vip3Aa* genes. (g) Rapid strip assay for protein expression. Lanes 1, 3, 5, 7, 9 and 11: Bar protein detection in pBC‐transformed BC‐1 to BC‐6 lines; Lanes 2, 4, 6, 8, 10 and 12: Cry1Ab protein detection in corresponding lines. (h) Southern blotting. pBC‐transformed LC05‐136 lines (BC‐2 to BC‐7, left); pBCV‐transformed lines (BCV‐2 to BCV‐7 and BCV‐8, right). M, marker; P, plasmid; −, WT negative controls; R, root DNA; S, stem DNA; L, leaf DNA. (i) ELISA analysis of pBC lines. (j) ELISA analysis of pBCV lines. (k) Herbicide tolerance and (l) insect resistance assays in transgenic sugarcane. (1) Stem inoculation with *Chilo infuscatellus* larvae; (2) larval weight measurement. (m) Average larval weight on different materials. WT, non‐transgenic control; BC‐OE, pBC‐transformed LC05‐136 line BC‐2; BCV‐OE, pBCV‐transformed LC05‐136 line BCV‐2. Data in (b), (e) and (m) were analysed using one‐way ANOVA, and data in (i) and (j) using Fisher's protected least significant difference (LSD) test. Error bars indicate standard deviations, grey dots represent individual replicates and different lowercase letters denote significant differences (*p* < 0.05).

Furthermore, transformation efficiency was assayed using six plant expression vectors (Figure 1c, Figure S3a,b). Specifically, callus tissues from all eight cultivars were infected with *Agrobacterium* carrying these constructs, followed by continuous herbicide selection (Figure 1d). Twenty‐one resistant plants or all if fewer than 21 were then randomly selected for DNA extraction from each transformation combination of different insect/herbicide‐resistant genes. PCR detection demonstrated all 100% positive rates in transgenic ROC22, LC05‐136 and ZT3 when using the pCCCC vector (containing CP4‐EPSPS as the selection marker). Across all eight cultivars, the positive rates ranged from 41.67% to 100% for transformants using different Bar‐containing vectors. Notably, the lowest efficiency (41.67%) was observed in ZT3, while LC05‐136 achieved a 100% positive rate, when transformed with the same pBC vector (Figure 1e and Table S2).

To evaluate the application potential of the transgenic plants developed above, the commercially important sugarcane cultivar LC05‐136 was selected. There were consistent results in transgenic plants detected by PCR detection of three target genes (*Bar*, *Cry1Ab*, and *Vip3Aa*) (Figure 1f, Figure S3c, Table S3) and rapid strip tests for Bar and Cry1Ab protein expression (Figure 1g, Table S3). Using a digoxigenin (DIG)‐labelled *Cry1Ab*‐specific probe, consistent hybridization patterns across root, stem and leaf were observed within individual lines, with distinct integration sites among different transformants (Figure 1h). ELISA and RT‐qPCR assays revealed high expression of Bar, Cry1Ab and Vip3Aa in all 12 transgenic lines (Figure 1i,j, Figure S3d–f). Herbicide resistance tests showed that non‐transgenic plants died at a concentration of 1.0 mg/mL glufosinate, while transgenic lines BC‐2 and BCV‐2 exhibited only mild growth inhibition even at 4.0 mg/mL (Figure 1k). Subsequently, stem tissues from non‐transgenic plants and transgenic lines (BC‐2, BCV‐2) were inoculated with *Chilo infuscatellus* larvae. After 10 days of dark rearing, non‐transgenic stems showed severe damage, while BC‐2 stems displayed significantly reduced but still visible feeding traces, and BCV‐2 stems remained nearly intact with minimal damage (Figure 1l). Larval weight measurements further disclosed average weights of 52 (±4.8) mg for insects fed non‐transgenic stems, compared to 18 (±3.4) mg and 5 (±1.9) mg for those fed BC and BCV transgenic stems, respectively (Figure 1m). Therefore, both single‐gene (*Cry1Ab*) and dual‐gene (*Cry1Ab* and *Vip3A*a) transformants significantly enhanced insect resistance, with the gene stacking showing superior efficacy. Meanwhile, herbicide tolerance and insect resistance assays were conducted on transgenic lines of all cultivars (Figure S4a,b).

Collectively, we successfully established a highly efficient and genotype‐independent transformation platform adaptable to diverse sugarcane genotypes. This addresses the long‐standing genotype‐dependent limitations in sugarcane genetic transformation, expanding the range of suitable recipient materials from traditional model genotypes to major commercial cultivars.

## Author Contributions

W.W., Y.Z., S.J., S.Z. and Y.Q. designed the experiments. W.W., D.W., W.Z., Y.Z., Z.Z., Y.H., T.S., L.S. and Q.W. performed the experiments and analysed the data. W.W., D.W., W.Z., Y.Z. and Z.Z. wrote the manuscript with critical inputs from the other co‐authors. Q.W., Y.Z., S.J., S.Z. and Y.Q. revised the manuscript. All authors approved the final manuscript.

## Supporting information


Figure S1–S4.

Table S1–S5.

## Data Availability

The data that supports the findings of this study are available in the Supporting Information of this article.
