# Supplementary material for: Highly Efficient and Genotype‐Independent Genetic Transformation System in Sugarcane
Source: Plant Biotechnol J. 2025 Dec 5;24(4):2241–3. doi: 10.1111/pbi.70486 (PMC13140336; doi:10.1111/pbi.70486)
Supplement: Supplementary file 1 — Figure S1–S4. [file PBI-24-2241-s001.docx]

**Supplemental materials and methods**

**Plant materials**

In this study, 8 elite sugarcane cultivars occupying more than 85% area cultivation in China were selected, with the characteristics of each cultivar as follows:

**ROC22, also known as Xintaitang22 (XTT22):** It was introduced from Chinese Taiwan to Guangxi in 1998. It is an excellent sugarcane cultivar with the largest cumulative planting area in China. As a representative cultivar of *Saccharum* hybrid (*S. Hybrid*), it is also an important parent in Chinese sugarcane breeding. Its planting area accounted for more than 85% of the total sugarcane planting area in China for 15 consecutive years, and over 90% of the fourth-generation sugarcane cultivars are its progenies (Wang et al., 2025a).

**Liucheng05136 (LC05-136):** A sugarcane cultivar bred by the research team led by Wenxiang Lu from Liucheng Sugarcane Research Center of Guangxi Zhuang Autonomous Region through hybridization, with ROC22 as the male parent and CP81-1254 as the female parent. By 2023, the cumulative national planting area of this cultivar has exceeded 1.67 million hectares, and in 2021, its planting area reached 0.30 million hectares, accounting for 38.30% of the total sugarcane planting area in China (Wu et al., 2024a). Its ranks among the top cultivars in both annual and cumulative planting areas nationwide.

**Guitang42 (GT42):** Bred by Lunwang Wang from Sugarcane Research Institute of Guangxi Academy of Agricultural Sciences, with ROC22 as the male parent and Guitang 92-66 as the female parent. With an annual planting area of over 0.28 million hectares, it is currently the largest sugarcane cultivar in China in terms of planting area and the main cultivar in Guangxi (Wu et al., 2025).

**Liucheng1541 (LC1541):** A sugarcane cultivar bred by Wenxiang Lu from Liucheng Sugarcane Research Center of Guangxi Zhuang Autonomous Region through hybridization, with Guiliu 2 as the male parent and Qiantang 4 as the female parent. This cultivar has excellent characteristics such as high yield, high sugar content, drought resistance, and cold resistance, and as a new-generation cultivar, it has broad prospects for popularization and application.

**Yunzhe08-1609 (YZ08-1609):** Bred by Hongming Xia and Peifang Zhao from Sugarcane Research Institute of Yunnan Academy of Agricultural Sciences, with Yuetang 00-236 as the male parent and Yunzhe 94-343 as the female parent. It features disease resistance, high yield, high sugar content, and suitability for mechanized harvesting. It is a major local cultivar with an annual planting area of over 0.12 million hectares in Yunnan Province (Wu et al., 2024b).

**Yunzhe05-51 (YZ05-51):** Bred by Jiayong Liu from Sugarcane Research Institute of Yunnan Academy of Agricultural Sciences, with ROC23 as the male parent and Yacheng 90-56 as the female parent. This cultivar exhibits advantages such as early maturity, high yield, high sugar content, strong drought resistance, and wide adaptability. Its annual planting area in Yunnan Province exceeds 40000 hectares, making it the second largest main cultivar in the region (Wu et al., 2024c).

**Zhongtang3 (ZT3):** Bred by Benpeng Yang from Institute of Tropical Bioscience and Biotechnology, Chinese Academy of Tropical Agricultural Sciences through hybridization, with Yuetang 99-66 as the male parent and ROC28 as the female parent. It has characteristics of high yield, high resistance to smut, and suitability for mechanized harvesting. As a new-generation sugarcane cultivar, it has good popularization potential.

**Huang pi guo zhe (HPGZ):** One yellow-rind fruit-type sugarcane (*Saccharum officinarum*) with the full Chinese name Huang pi guo zhe. It is one of the original parents of modern sugarcane cultivars, with a gene contribution rate of 78%, and is an important donor of high-yield and high-sugar gene resources. Establishing a genetic transformation system for this cultivar will help clarify the molecular mechanism of sugar accumulation in sugarcane and explore key genes regulating high sugar content.

**Callus Induction Process**

Healthy sugarcane plants grown in a greenhouse or field were selected. Apical shoot tips were excised and used as explants. The outer green leaves and leaf sheaths were removed to expose the young and white inner leaf tissue surrounding the apical meristem (Bower and Birch 1992). The explants were rinsed in sterile distilled water and then subjected to sequential surface sterilization: first, immersion in 75% ethanol for 30 seconds, followed by three times washes with sterile distilled water; second, transfer to a 0.1% mercuric chloride solution (or sodium hypochlorite solution, NaClO) for 10 min, followed by rinsing 5-6 times with sterile distilled water (1-2 min per rinse). Finally, surface moisture was removed using sterile filter paper.

Using a sterile scalpel blade, the explants were transversely sectioned into thin slices approximately 0.1 mm thick. These slices were inoculated onto callus induction medium. Ten explant slices were inoculated per culture dish. Cultures were incubated at 28°C in the dark. The medium was replaced with fresh callus induction medium every 15 days for subculturing. This subculture process was repeated for 1 to 4 cycles, with the total culture period maintained between 30 and 75 days. The formula of tissue culture medium in each stage of sugarcane genetic transformation is slightly modified from Murashige and Skoog (1962) and shown in Table S5.

**Determination of the optimal 2,4-D concentration for callus induction**

According to the sterilization and processing methods described above, explant slices from different sugarcane cultivars were prepared. These slices were then inoculated onto callus induction medium supplemented with varying concentrations of 2,4-D: 0.5, 1.0, 1.5, 2.0, 2.5, 3.0, 3.5, and 4.0 mg/L. Inoculated culture dishes were incubated at 28°C under continuous darkness. Every 15 days, the callus tissues were excised and subdivided, then transferred at a 1:3 ratio onto fresh callus induction medium containing the same respective 2,4-D concentration for subculturing. This subculture process was repeated three times, resulting in a total culture period of 60 days.

After subculture, callus tissues from each treatment (each cultivar at each 2,4-D concentration) were transferred onto differentiation medium. Each treatment was set up with 50 callus pieces (10 pieces × 5 dishes). Differentiation cultures were maintained in a growth chamber under the following conditions: a 14-hour photoperiod, a day temperature of 35°C, a night temperature of 30°C, for a period of 20 days. At the end of the differentiation culture period, callus tissues were photographed for documentation. Differentiation rates of each callus were quantified using a 5-point scoring system: 0 (no differentiation), 1 (differentiation rate < 25%), 2 (25% ≤ differentiation rate < 50%), 3 (50% ≤ differentiation rate < 75%), and 4 (differentiation rate ≥ 75%). The average differentiation score was calculated for each treatment. The 2,4-D concentration corresponding to the treatment group with the highest score was designated as the optimal concentration for callus induction and subculture.

**Determination of optimal subculturing frequency for callus induction**

Callus tissues from different sugarcane cultivars, obtained after 1 to 4 subculturing cycles (at 15-day intervals; total culture periods ranging from 15 to 75 days) at their optimal 2,4-D concentrations, were transferred to differentiation medium. For each subculturing treatment, 50 callus pieces (10 pieces × 5 dishes) were inoculated per cultivar. Differentiation conditions were as described above. After differentiation, callus tissues were photographed. Differentiation rates were scored using the 5-point scale described above. The average differentiation score was calculated for each cultivar at each subculturing frequency. The subculturing frequency yielding the highest average score was identified as the optimal for that cultivar.

**Statistics of regeneration efficiency for each cultivar**

Compared callus induction and regeneration rates across cultivars under their respective optimal 2,4-D concentrations and subculture durations. Specifically, using the optimized system (optimal 2,4-D concentration and subculturing frequency for each cultivar), tissue culture was performed using 3 independent explants per cultivar according to the following procedure: first, callus induction culture (15 days), followed by subculture proliferation (30-45 days), then transfer to differentiation medium for differentiation and proliferation (20 days), and finally, the differentiated seedlings were inoculated onto rooting medium for two consecutive 20-day cycles. The number of successfully rooted plants developing into complete plantlets was recorded for each cultivar. Regeneration efficiency was calculated as: Number of regenerated plantlets/Number of initial explants.

**Information on insect-resistant and herbicide-tolerant plant expression vectors**

All vectors were introduced into *Agrobacterium* tumefaciens strain EHA105 using the freeze-thaw method. The transformed bacterial solutions were aliquoted and stored at -80℃ for later use. The plant expression vectors used in this study included:

**pBar+Cry1Ab (pBC):** Contains the *Bar* herbicide-resistance selectable marker gene and the single *Cry1Ab* insect-resistant gene. Constructed by our laboratory.

**pBar+Cry1Ab+Vip3Aa (pBCV):** Contains the *Bar* herbicide-resistance gene and the dual *Cry1Ab* and *Vip3Aa* insect-resistant genes. Constructed by our laboratory.

**pBar+Cry9Cb (pBC_9_):** Contains the *Bar* gene and the single *Cry9Cb* insect-resistant gene. Kindly provided by the research team of Professor Zhang Jie from Institute of Plant Protection, Chinese Academy of Agricultural Sciences.

**pBar+Cry9Cb+Vip3Aa (pBC_9_V):** Contains the *Bar* herbicide-resistance gene and the dual *Cry9Cb* and *Vip3Aa* insect-resistant genes. Kindly also provided by Professor Zhang Jie’s team.

**pBar+CP4-EPSPS+Cry1Ab (pBCC):** Contains the dual *Bar* and *CP4-EPSPS* herbicide-resistance genes, and the single *Cry1Ab* insect-resistant gene. Kindly provided by Longping Biotechnology Hainan Co., Ltd.

**pCP4-EPSPS+Cry1Ab+Cry2Ab+Cry1Fa** **(pCCCC):** Contains *CP4-EPSPS* herbicide-resistance gene, and *Cry1Ab*, *Cry2Ab* and *Cry1Fa* insect-resistant genes. Kindly provided by Longping Biotechnology Hainan Co., Ltd.

**Preparation of *Agrobacterium* infection solution**

*Agrobacterium* glycerol stocks containing each expression vector were retrieved from -80°C storage. Strains were streak-plated onto YEP solid medium supplemented with corresponding antibiotics. Plates were inverted and incubated at 28°C for 2-3 days. Single colonies were picked and inoculated into liquid YEP medium containing corresponding antibiotics, then incubated overnight at 28°C, 200 rpm. 2 µL of each overnight culture was used as a PCR template with vector-specific primers. Correct/positive strains were confirmed by agarose gel electrophoresis. PCR-positive cultures were diluted 1:40 (e.g., 10 µL culture into 400 µL sterile water) and spread-plated onto YEP solid medium containing 100 µM acetosyringone. Plates were incubated overnight at 28°C. Bacterial growth was scraped from the plate surface and resuspended in immersion culture medium (Table S5). The suspension’s OD_600_ was adjusted to 0.6. The suspension was incubated in the dark at 28°C with shaking at 100 rpm for 1-2 hours to obtain the *Agrobacterium* infection solution (Wang et al., 2017a).

**Genetic transformation and screening of resistant plants**

The calluses with high regeneration rate induced from various sugarcane cultivars were placed on sterile filter paper. After the surface moisture was dried, they were transferred to an Erlenmeyer flask containing 50 mL of *Agrobacterium* infection solution, followed by shaking culture at 28 ℃ and 100 rpm in the dark for 10 min. Subsequently, the Erlenmeyer flask was put into an ultrasonic cleaner and treated at maximum power for 2 min; the infection solution was discarded, and 50 mL of fresh *Agrobacterium* infection solution was added again, followed by treatment under a vacuum condition of -0.08MPa for 5 min; then, the calluses were transferred to a constant temperature shaker for dark shaking culture at 28℃ and 100 rpm for 10 min (Dong et al., 2014). After the treatment, the calluses were filtered out, the excess bacterial solution was blotted with sterile filter paper, and air-dried at room temperature in an ultra-clean workbench for 30 min.

The infected calluses were inoculated into the co-culture medium and co-cultured at 21℃ in the dark for 2-3 days; then the calluses were transferred to the recovery medium for recovery culture at 28℃ in the dark for 7 days; subsequently, they were transferred to the callus screening medium for screening culture at 28℃ in the dark for 30 days; the screened calluses were transferred to the differentiation screening medium for differentiation culture under the conditions of 14 h/d light, day temperature of 35℃ and night temperature of 30℃ for 20 days; the differentiated resistant plants were transferred to the rooting screening medium for rooting culture under the same light and temperature conditions for 40 days, and finally transplanted to the greenhouse (Wang et al., 2025b) (Table S5).

**PCR detection of resistant plants**

DNA was extracted from the leaves of resistant plants using the CTAB method. PCR amplification was performed with Taq enzyme (Vazyme, P131-02) using specific primers for the target genes in each plant expression vector (Figure S3b and S3c). The reaction program was as follows: pre-denaturation at 95℃ for 5 min, denaturation at 95℃ for 30 s, annealing at 58℃ for 30 s, extension at 72℃ for 1 min, with 35 cycles; final extension at 72℃ for 10 min. The products were detected by 1.5% agarose gel electrophoresis. For different experiments, specific primers from Table S4 were used. To calculate the transformation efficiency in eight sugarcane varieties, transgenic plants were detected using the *Bar* and *CP4-EPSPS* primer pairs. The transformation efficiency was calculated as: Number of PCR-positive plants/Total number of resistant plants × 100%. Meanwhile, the *Bar*, *Cry1Ab*, and *Vip3Aa* primers were used to detect target genes in the confirmed transgenic plants of variety LC05-136.

**Rapid test strip detection of transgenic plants**

Rapid detection test strips for *Bar* and *Cry1Ab* genes (Genesino Biological S&T Development Co. Ltd., Dalian, China) were used to detect the expression of exogenous proteins following the kit instructions. Approximately 0.2 g of young leaves from the plants was taken, ground, added with 1 mL of extraction buffer, vortexed to mix, and left to stand at room temperature for 5 min. The test strip was immersed in the supernatant, and the color development of the test line and control line was observed within 5-10 min to determine whether the protein expression was positive or negative.

**Southern blotting of transgenic plants**

After the plants were transplanted into the greenhouse or field, DNA (30 μg) was extracted from the roots, stems, and leaf tissues of transgenic sugarcane plants that were positive in both the target gene PCR and test strip detection. The DNA was digested with the restriction enzyme *Hind* III. After separating the digested products by agarose gel electrophoresis, they were transferred to a nylon membrane. A specific fragment of the target gene amplified by PCR, labeled with digoxigenin (DIG), was used as a probe. Southern blotting was performed according to the instructions of the DIG High Prime DNA Labeling and Detection Starter Kit II (Roche). The copy number of transgenic plants was analyzed by the number of hybrid bands, and the consistency of the insertion copy number of exogenous genes in roots, stems, and leaves of transgenic plants was analyzed.

**RT-qPCR of transgenic plants**

Total RNA was extracted from the leaves of transgenic sugarcane plants and reverse-transcribed into cDNA using the HiScript II Q RT SuperMix for qPCR kit (Yeasen, Shanghai, China) after quality assessment. RT-qPCR was performed using the ChamQ Blue Universal SYBR qPCR Master Mix (Vazyme, Nanjing, China) according to the manufacturer’s instructions, with non-transformed plants as controls and cDNA as templates. Primers used for detection are listed in Table S4. Relative expression levels of target genes were calculated using the 2^⁻ΔΔCt^ method, with the glyceraldehyde-3-phosphate dehydrogenase (*GAPDH*) as the internal reference gene.

**ELISA assay of transgenic plants**

Stem tissue (100 mg) was excised from transgenic lines that tested positive for PCR detection of the *Bar*, *Cry1Ab* and *Vip3Aa* genes. The tissue was ground into a homogenate using a tissue grinder (MM400, RETSCH, Germany), and crude protein was extracted using PBST buffer. Standard curves were established using the standards provided in the ELISA kits (Bar, MM-6264601; Cry1Ab, MM-6272201, Vip3Aa, MM-1241PL1, Jiangsu Meimian industrial Co., Ltd). Quantitative detection of protein was performed based on the absorbance (OD value) of samples from different lines at a wavelength of 450 nm.

**Indoor herbicide resistance bioassay of transgenic plants**

Transgenic sugarcane plants were transplanted into a greenhouse (temperature 30 ± 2°C, light 14 h/d). When the plants grew to the 6-8 leaf stage, tillers of plants with consistent growth were selected for treatment. Transgenic *Bar* gene plants were sprayed with 0, 1.0, 2.0, 3.0, 4.0, and 5.0 mg/mL glufosinate-ammonium and Transgenic *CP4-EPSPS* gene plants were sprayed with 0, 5.0, 10.0, 15.0, 20.0, 25.0 mg/mL glyphosate. Non-transgenic plants at the same growth stage were used as negative controls. Seven days after spraying, phenotypic changes such as leaf yellowing and wilting were observed, recorded, and photographed to evaluate the herbicide resistance effect (Wang et al., 2024).

**Indoor insect resistance bioassay of transgenic plants**

Stem tissues were cut and placed in a closed dark box containing moist filter paper. Then 3rd-instar *Chilo infuscatellus* larvae (provided by Dr. Xiankun Shang from Guangxi Academy of Agricultural Sciences, reared on artificial diet indoors) were introduced into each box. After 10 days, the body weight of surviving larvae was weighed using an electronic balance, the average weight gain rate of the larvae was calculated, and the differences in insect resistance were compared (Wang et al., 2017b).

**Supplemental References**

Bower, R., Brich R.G. (1992) Transgenic sugarcane plants via microprojectile bombardment. *Plant J.* **2**, 409–416.

Dong, S.J., Delucca, P., Geijskes, R. J., Ke, J., Mayo, K., Mai, P., Sainz, M., Caffall, K., Moser, T., Yarnall, M., *et al*. (2014). Advances in *Agrobacterium*-mediated sugarcane transformation and stable transgene expression. *Sugar Tech.* **16**, 366–371.

Murashige, T., Skoog, F. (1962) A revised medium for rapid growth and bioassays with tobacco tissue cultures. *Plant Physiol.* **15**, 473–497.

Wang, H., Pan, Y.B., Wu, M.X., Liu, J.H., Yang, S.W., Wu, Q.B., Que, Y.X. (2025a) Sugarcane genetics: Underlying theory and practical application. *Crop J*. **13**, 328–338.

Wang, D., Gou, Y., Yi, C., Li, Z., Wang, W., Lin, P., Wang, W. *et al*. (2025b) ScWRKY2: a key regulator for smut resistance in sugarcane, *Plant Biotechnol. J.* **23**, 3667–3681.

Wang, W., Yang, B., Feng, C., Wang J., Xiong, G., Zhao, T., Zhang, S. (2017a) Efficient sugarcane transformation via *bar* gene selection. *Tropical Plant Bio*. **10**, 77–85.

Wang, W.Z., Yang, B.P., Feng, X.Y., Cao, Z.Y., Feng, C.L., Wang, J.G., *et al.* (2017b) Development and characterization of transgenic sugarcane with insect resistance and herbicide tolerance. *Front. Plant Sci.* **8**, 1535–1545.

Wang, W., Talha, J., Shen, L., Sun, T., Yang, B., Zhang, S. (2024) Establishment of an efficient sugarcane transformation system via herbicide-resistant CP4-EPSPS gene selection. *Plants*. **13**, 852.

Wu, Q., Li, Z., Lu, W., Liang, F., Zhang, Y., and Que, Y. (2024a) LC05-136 originates from ROC22, green arising from blue and surpassing blue. *Trop. plants.* **3**, e024.

Wu, Q., Li, A., Zhao, P., Xia, H., Zhang, Y., and Que, Y. (2024b) Theory to practice: a success in breeding sugarcane variety YZ08-1609 known as the King of Sugar. *Front. Plant Sci.* **15**, 1413108.

Wu, Q., Li, A., Liu, J., Zhao, Y., Zhao, P., Zhang, Y., and Que, Y. (2024c) Sugarcane variety YZ05-51 with high yield and strong resistance: breeding and cultivation perspectives. *Trop. plants.* **3**, e017.

Wu, Q., Li, Z., Li, A., Wang, L., Tang, S., Zhou, H., Zhang, Y., and Que, Y. (2025) Sugarcane varieties GT 42 and GT 44, not a flash in the pan but the flag in the ship, *Sugar Tech.* **27**, 627–634.

**Supplemental Figures**


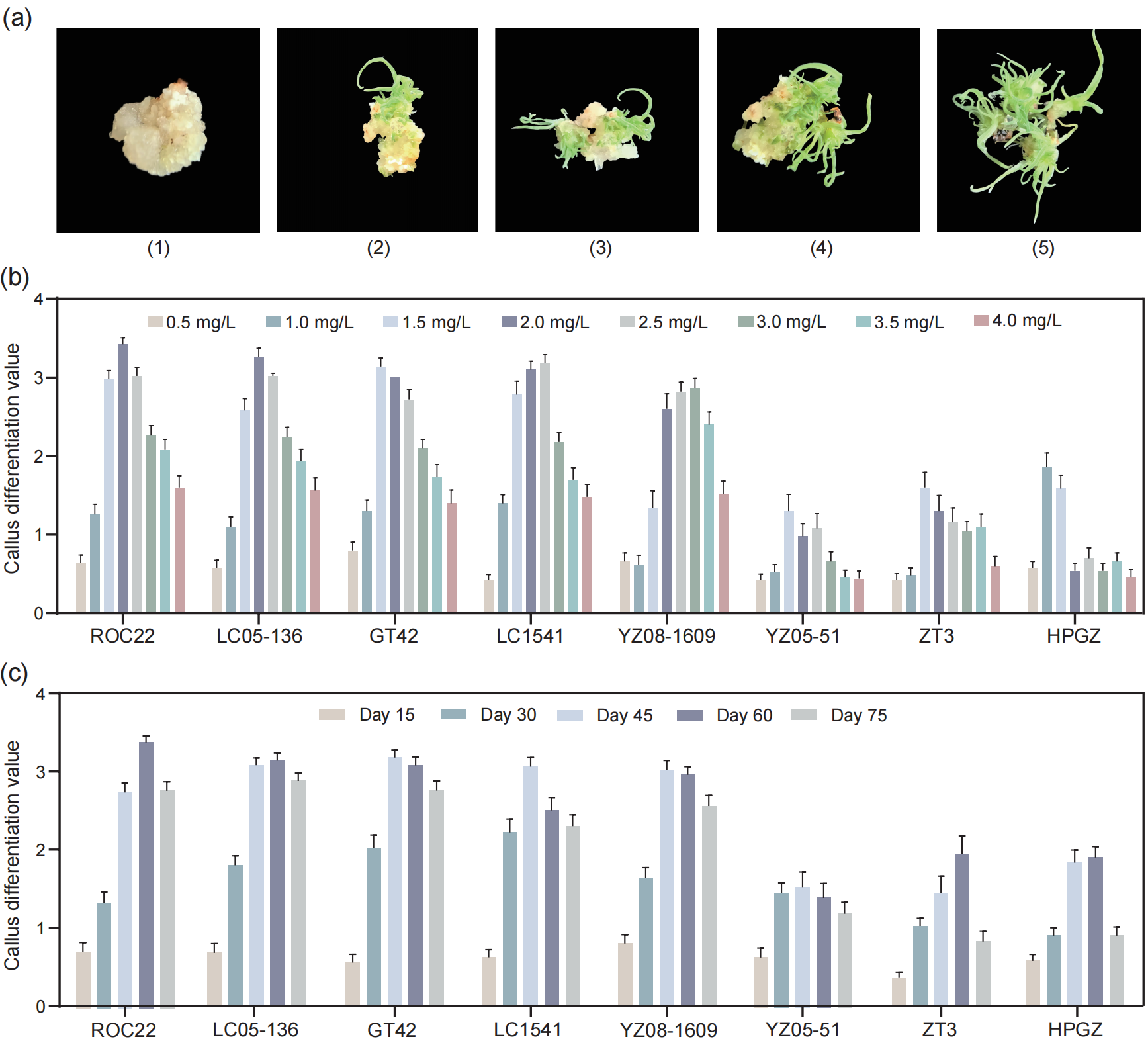


**Figure S1** **Auxin concentration and callus induction for eight sugarcane cultivars.** (a) Classification of callus differentiation into five grades. (1) callus unable to ‌redifferentiation scored 0; (2) callus with not more than 25% surface cell redifferentiation scored 1; (3) callus with 25%-50% surface cell redifferentiation scored 2; (4) callus with 51%-75% surface cell redifferentiation scored 3; (5) callus with more than 75% surface cell redifferentiation scored 4. (b) Callus differentiation value of eight sugarcane cultivars under different 2,4-D concentrations. (c) Callus differentiation value of eight sugarcane cultivars at different induction durations.


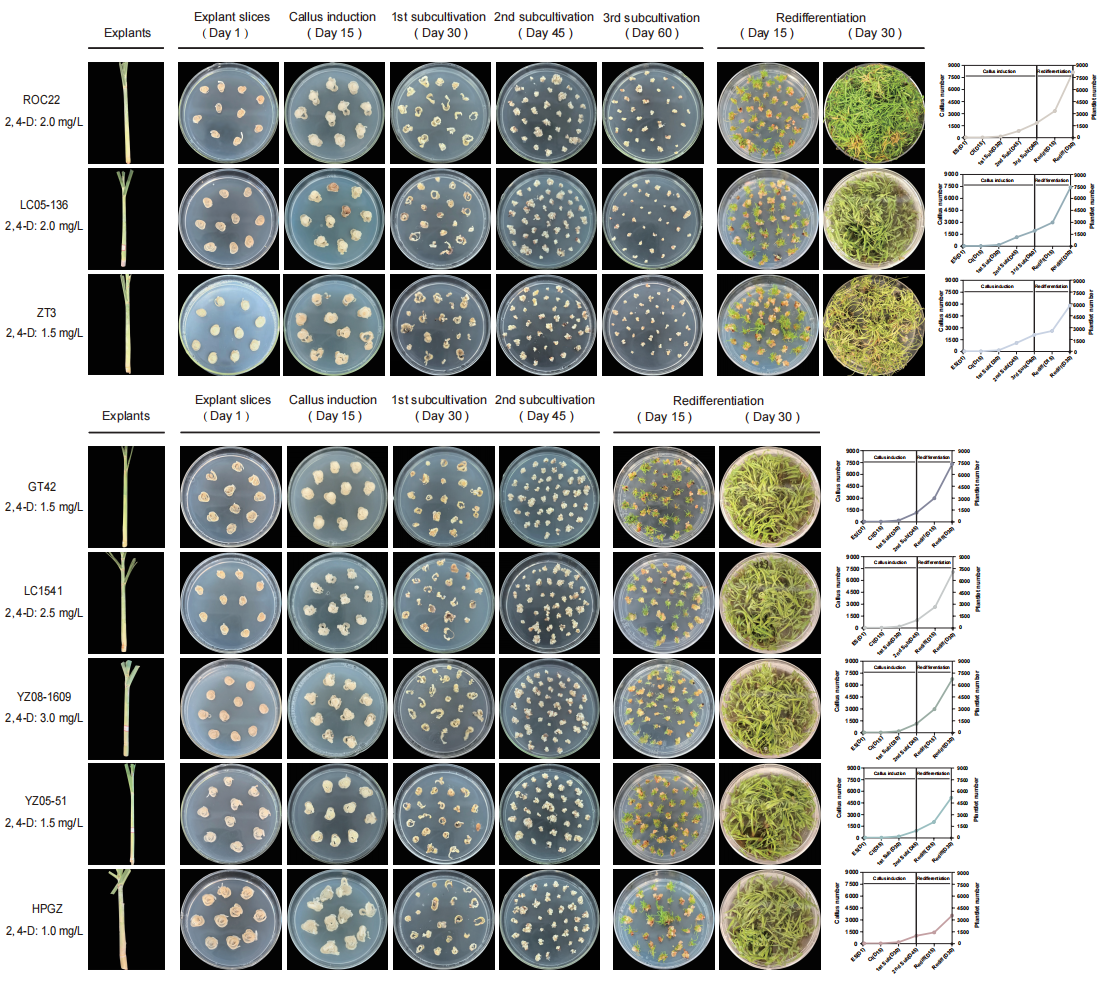


**Figure S2 Somatic cell propagation system for eight cultivars.** The growth and differentiation status of callus cultured under optimal auxin concentrations and subculture cycles for each cultivar. The line graphs illustrated the proliferation rates of callus during both the callus induction and redifferentiation phases for each cultivar. ES (D1), Explant slices (Day 1); CI (D15), Callus induction (Day 15); 1st Sub (D30), First subcultivation (Day 30); 2nd Sub (D45), Second subcultivation (Day 45); 3rd Sub (D60), Third subcultivation (Day 60); Rediff (D15), Regeneration (Day 15); Rediff (D30), Regeneration (Day 30).


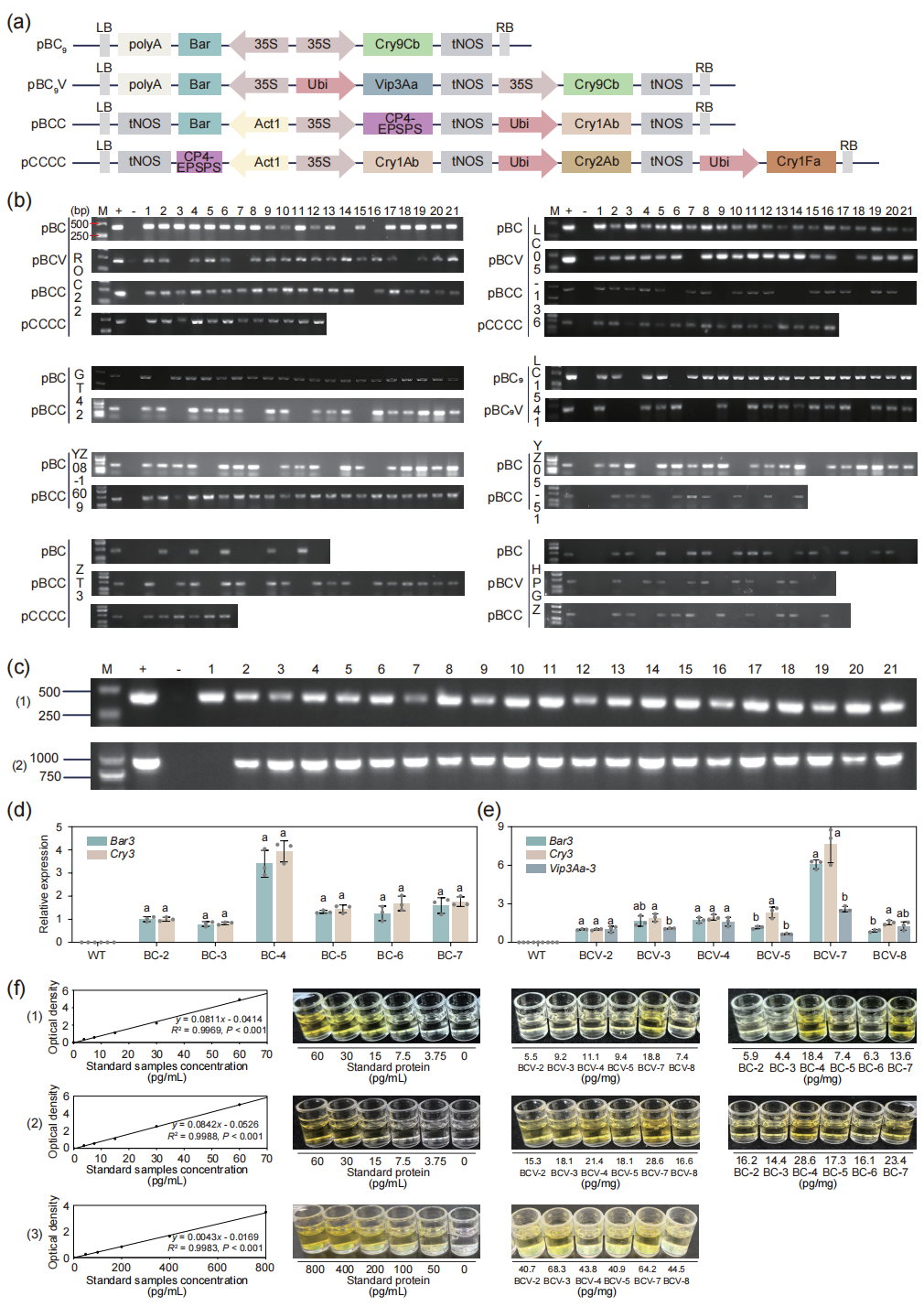


**Figure S3** **Construction of transgenic vectors and molecular analysis of transgenic sugarcane progeny.** (a) Four over-expression vectors used in this experiment. (b) PCR detection of genetically transformed eight sugarcane cultivars using different over-expression vectors. The transgenic lines with the pCCCC vector were identified using primers for the *CP4-EPSPS* selectable marker gene, while lines with the other vectors were identified using primers for the *Bar* gene. (c) PCR detection of transgenic LC05-136 plants carrying the pBC vector. (1) detection of the *Bar* gene by PCR; (2) detection of the *Cry1Ab* gene by PCR. (d) RT-qPCR assay of *Bar* and *Cry1Ab* gene transcriptional level of pBC transgenic lines. (e) RT-qPCR assay of *Bar*, *Cry1Ab* and *Vip3Aa* gene transcriptional level of pBCV transgenic lines. (f) Quantification of Bar, Cry1Ab and Vip3Aa proteins in sugarcane transgenic lines harboring pBC and pBCV overexpression constructs. (1) bar protein levels were determined by ELISA. Left, corresponding standard curve (0-70 pg/mL); Right, protein expression in 12 independent pBC and pBCV transgenic lines. (2) Cry1Ab protein levels were determined by ELISA. Left, corresponding standard curve (0-70 pg/mL); Right, protein expression in 12 independent pBC and pBCV transgenic lines. (3) Vip3Aa protein levels were determined by ELISA. Left, corresponding standard curve (0-800 pg/mL); Right, protein expression in 6 independent pBCV transgenic lines. Data in (d) and (e) were analyzed using Fisher’s protected least significant difference (LSD) test. Error bars indicate standard deviations, gray dots represent individual replicates, and different lowercase letters denote significant differences (*P* < 0.05).


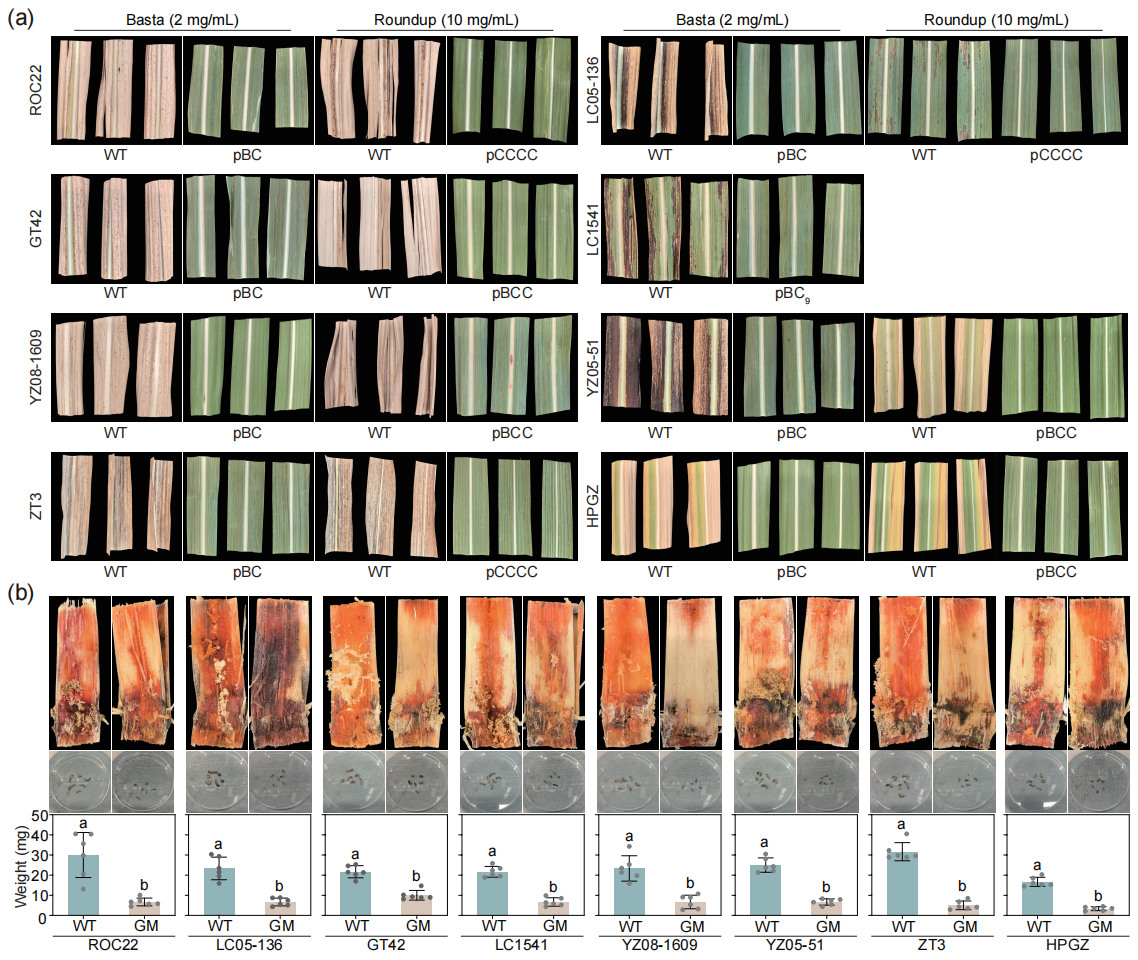


**Figure S4 Herbicide tolerance and insect resistance assays in transgenic plants of eight sugarcane cultivars.** (a) Herbicide tolerance assay by spray 2.0 mg/mL glufosinate or 10 mg/mL glyphosate on eight sugarcane cultivars which integrated with herbicide resistant *Bar* gene or *CP4-EPSPS* gene. (b) Insect resistant assay of ELISA positive transgenic shoots from eight cultivar. Data were analyzed using one-way ANOVA. Error bars indicate standard deviations, gray dots represent individual replicates, and different lowercase letters denote significant differences (*P* < 0.05).
